# Implementation of a Specialty-Focused Musculoskeletal Service in a Medical Student-Run Free Clinic in Puerto Rico

**DOI:** 10.7759/cureus.107621

**Published:** 2026-04-23

**Authors:** Gonzalo F Del Rio Montesinos, Andrea Fabregas, Andrea Vazquez

**Affiliations:** 1 Orthopaedics, Universidad Central del Caribe, Bayamon, USA

**Keywords:** medically underserved areas, musculoskeletal assessment, student-run clinic, underserved populations, urban and rural community

## Abstract

Musculoskeletal (MSK) conditions are a leading cause of pain, disability, and functional limitation worldwide and disproportionately affect underserved populations. Student-run free clinics frequently provide essential primary care services but often lack the specialty capacity needed to address MSK-related complaints. This study describes the implementation of a specialty-focused MSK clinic within a community-based student-run free clinic in Puerto Rico.

A specialty-focused MSK clinic was implemented within an existing student-run free clinic in Puerto Rico from April 2025 through March 2026 using an interdisciplinary model that integrated volunteer orthopedic surgeons and physical medicine and rehabilitation specialists into the clinic’s care structure. During this period, a total of 54 patients attended the clinic. The clinic emphasized functional assessment, patient education, and management planning within a trusted community-based setting. Evaluation relied on descriptive clinic metrics, patient-reported presenting complaints, reflective practice through post-clinic debriefings, and informal feedback from patients and volunteers to guide iterative refinement.

During the study period, MSK pain, prior injuries, and mobility-related concerns were among the most common patient-reported complaints. Implementation of the MSK clinic expanded the clinic’s capacity to evaluate and manage chronic and acute MSK conditions that had previously been under-addressed because of limited specialty access. Early observations included improved identification of MSK conditions, greater emphasis on pain management and functional restoration, increased patient understanding of care plans, and strong interdisciplinary collaboration among specialty and primary care volunteers.

Integrating specialty MSK services into a student-run free clinic is a feasible approach to addressing an important service gap in underserved populations. This model highlights the value of interdisciplinary collaboration and may be adaptable to similar resource-limited clinical settings seeking to strengthen community-based specialty care and address MSK health needs.

## Introduction

Musculoskeletal (MSK) conditions are a leading cause of pain, disability, and functional limitation worldwide. Among Puerto Ricans, these conditions are particularly prevalent. Data from the U.S. Centers for Disease Control and Prevention indicate that 21.8% of Puerto Ricans have doctor-diagnosed arthritis, the highest prevalence among Hispanic subgroups [1]. Nearly half of those with arthritis experience activity limitations and 44.1% report severe joint pain [1]. High-impact chronic pain is more common among Puerto Ricans compared with other Hispanic ancestry groups [2], and older adults report substantial difficulty with activities of daily living [3]. Local surveys suggest that approximately 26% of adults in Puerto Rico have medically diagnosed arthritis [4]. This burden persisted after Hurricane Maria [5], highlighting the compounded effects of structural vulnerability and limited access to specialty care. Student-run free clinics are community-based healthcare settings that provide no-cost or low-cost services to underserved populations while offering supervised clinical training opportunities for medical students. These clinics are typically staffed by students working under licensed physician supervision and often focus on primary and preventive care. As a result, they play an important role in addressing healthcare gaps, but many have limited access to specialty services such as MSK evaluation and management.

In our community-based student-run free clinic, during the period from April 2025 through March 2026, MSK pain, prior injuries, and mobility-related complaints represented common patient-reported concerns. Survey-based assessment within this clinical setting further supported the need for targeted MSK services. Despite this demand, the clinic lacked consistent access to orthopedic and physical medicine and rehabilitation (PM&R) specialists. This gap reflects a broader limitation of student-run free clinics, which often lack consistent access to specialty care, particularly in MSK health, despite high patient demand. This need is consistent with prior work from our clinic, which identified MSK conditions as a major unmet need within the population served [6].

## Technical report

To address this need, we implemented an interdisciplinary MSK clinic embedded within the existing student-run free clinic from April 2025 through March 2026. During this period, a total of 54 patients attended. Guided by principles of community-oriented primary care, the clinic integrated volunteer orthopedic surgeons and PM&R physicians into the established care structure. This model emphasized comprehensive functional assessment, patient education, and shared decision-making within a familiar community setting. Students and primary care volunteers collaborated with specialists during each clinic session, enabling a holistic evaluation of MSK conditions and individualized management plans.

Workflow design prioritized sustainability and reproducibility. Standardized intake forms captured pain characteristics, functional limitations, and goals. After individual assessments, interdisciplinary discussions facilitated consensus on diagnosis and management. Care plans included conservative management, referrals for imaging when necessary, and patient-specific rehabilitation exercises. Documentation templates ensured continuity of care and facilitated follow-up. Educational materials in Spanish and English were developed to promote self-management and understanding of MSK conditions. Volunteer coordination and scheduling systems were established to maximize specialist availability.

Table 1 summarizes the standardized workflow used to implement the MSK service within the student-run free clinic. Each step was designed to promote consistency, interdisciplinary collaboration, and continuity of care, from initial intake through follow-up and evaluation. Together, these workflow components supported a structured and reproducible approach to MSK assessment and management in a resource-limited setting and contributed to improved identification of MSK conditions, enhanced patient understanding of care plans, and greater emphasis on functional management.

**Table 1:** Standardized workflow steps for implementation of the musculoskeletal service within the student-run free clinic, from intake through follow-up and evaluation. PM&R: Physical medicine and rehabilitation

Workflow step	Description
Patient intake	Use standardized intake forms to capture pain characteristics, functional limitations, and patient goals.
Functional assessment	Students and specialists perform comprehensive physical examination and functional evaluation to determine diagnoses and assess mobility.
Interdisciplinary discussion	Students, primary care volunteers, orthopedic surgeons and PM&R physicians discuss each case to reach a consensus on diagnosis and management.
Management and education	Develop individualized care plans combining conservative management, referrals for imaging if necessary, rehabilitation exercises, and education in Spanish and English.
Follow‑up and evaluation	Document care, schedule follow‑ups, and collect feedback and patient‑reported outcomes to monitor progress and inform continuous improvement.

Implementation outcomes, representing the primary results of this initiative, focused on improving access to MSK care and enhancing educational experiences for students. Early observations indicated better identification and management of chronic and acute MSK conditions, increased patient understanding of diagnoses and treatment options, and greater attention to pain management and functional restoration. Medical students gained exposure to interdisciplinary MSK care, underscoring the potential role of specialty services in addressing unmet MSK care needs in underserved settings.

Evaluation used pragmatic methods suited to a resource-limited setting and was intentionally focused on descriptive implementation outcomes rather than formal statistical analysis. Basic descriptive measures, including patient volume and distribution of presenting complaints, were used to characterize clinic activity. Post-clinic debriefings with students and volunteers facilitated iterative improvement. Informal patient feedback provided insights into accessibility and perceived quality of care. As the clinic expanded, evaluation shifted toward functional outcomes, including plans to incorporate structured patient-reported outcome measures of pain and mobility and to monitor continuity of care. These findings are descriptive in nature and reflect observed trends within the clinic rather than formal comparative or inferential analysis.

**Figure 1:**
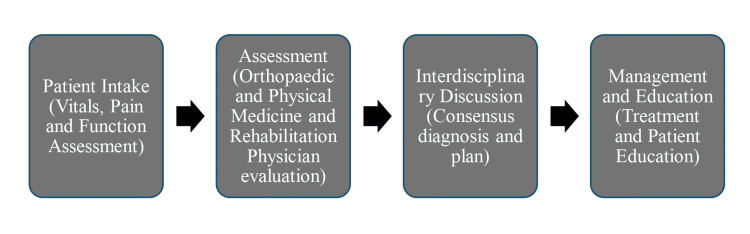
Interdisciplinary clinic workflow illustrating intake, assessment, consensus, and management steps.

Figure 1 illustrates the interdisciplinary workflow of the MSK clinic, highlighting the progression from patient intake and functional assessment to team-based discussion and individualized management planning. This figure visually reinforces how the clinic integrated specialty input into an existing student-run free clinic model and how each stage contributed to coordinated, function-centered care and the observed improvements in patient evaluation and management. 

Ethical considerations

This manuscript describes a quality improvement and implementation initiative conducted within routine clinical care at a student-run free clinic. No identifiable patient information is included. As this work does not meet the definition of human subjects research, Institutional Review Board approval was not required, and the requirement for informed consent was waived.

## Discussion

This technical report describes the implementation of a specialty-focused MSK service within a student-run free clinic serving an underserved population in Puerto Rico. The findings highlight the feasibility of integrating specialty services into community-based settings and underscore the importance of addressing unmet MSK care needs in underserved populations. These observations are consistent with prior work by McQuillan et al., who demonstrated that integrating a dedicated MSK clinic within a student-run free clinic enabled the management of diverse MSK conditions while maintaining high levels of student satisfaction, with 95% of participants reporting positive educational experiences [7]. Their model also emphasized the value of structured referral systems, interdisciplinary supervision, and longitudinal follow-up, all of which similarly informed the design of our workflow. In addition, their findings identified knee and back pain as among the most common presenting complaints, further reinforcing the relevance of targeted MSK services in underserved populations [7].

This need was further supported by a separate IRB-approved survey-based assessment conducted in the same clinical setting, in which 48 of 62 respondents reported more than one MSK limitation. Together, these findings reinforce the high burden of unmet MSK needs within this underserved setting.

Similarly, Vomer et al. described a student, resident, and attending-led MSK clinic integrating physical therapy and osteopathic manipulative treatment, demonstrating significant improvements in student knowledge and confidence as well as patient-reported pain and mobility outcomes [8]. Their study showed measurable gains in MSK knowledge scores and confidence following participation, supporting the educational value of hands-on, interdisciplinary models [8]. Their structured clinic design, which included standardized intake forms, functional outcome measures, and iterative follow-up, also parallels the reproducible framework implemented in our model and reinforces the importance of standardized processes in achieving both educational and clinical impact [8]. Taken together, these prior studies support the scalability of interdisciplinary MSK services within student-run clinics as an effective strategy to improve both access to care and medical education.

Within our clinic, the interdisciplinary model played a central role in implementation. Collaboration between orthopedic surgeons, PM&R specialists, and primary care providers allowed for more comprehensive evaluation of MSK conditions and supported a function-oriented approach to care. Embedding specialty services within a trusted community setting may help reduce barriers associated with traditional referral pathways, which are often difficult to access for underserved populations. The emphasis on function-centered care represented a particularly important strength of this model. Rather than focusing only on symptom relief, the clinic prioritized mobility, independence, and quality of life. This broader approach is especially relevant in underserved settings, where untreated MSK conditions can have lasting effects on daily function, work capacity, and overall well-being.

At the same time, the implementation process highlighted several challenges that are common in resource-limited and volunteer-driven settings. These included dependence on specialist availability, limited diagnostic resources, and difficulty maintaining continuity of care over time. Addressing such barriers requires structured workflows, clearly defined team roles, and sustainable systems for follow-up and coordination.

This study has some limitations. It represents a descriptive report from a single clinic and does not include formal comparative outcome measures. Evaluation relied primarily on descriptive metrics and informal feedback rather than standardized patient-reported outcomes. In addition, because this report reflects an early phase of implementation, it does not allow assessment of long-term clinical or educational impact. Future directions include incorporating standardized functional outcome measures, improving longitudinal follow-up, and expanding interdisciplinary services. Further study is needed to evaluate the long-term effectiveness, reproducibility, and scalability of similar models in other underserved settings. Additional clinics are also planned in the future to further expand access to specialty MSK care and strengthen longitudinal evaluation of this implementation model.

## Conclusions

Implementation of a specialty-focused MSK service within a student-run free clinic in Puerto Rico was feasible and addressed an important service gap in an underserved population. By integrating orthopedic and PM&R expertise into an existing community-based model, the clinic expanded access to specialty-informed, function-centered care while also enhancing student exposure to interdisciplinary MSK evaluation and management. This approach may offer a practical and reproducible framework for other resource-limited settings seeking to strengthen community-based specialty care and address MSK health needs.
